# Keypoint-Based Forest Musk Deer Behavioral Recognition Method

**DOI:** 10.3390/ani16111594

**Published:** 2026-05-23

**Authors:** Dequan Guo, Chuankang Chen, Chengli Zheng, Zhenyu Wang, Dapeng Zhang, Dening Luo

**Affiliations:** 1 School of Automation, Chengdu University of Information Technology, Chengdu 610225, China; 2 Sichuan Institute of Musk Deer Breeding (Sichuan Institute for Drug Control), Chengdu 611845, China; 3 Sichuan Chuanrongda Technology Co., Ltd., Chengdu 610041, China

**Keywords:** musk deer, keypoint detection, improved YOLOv8-Pose, behavioral recognition, real-time monitoring

## Abstract

The traditional monitoring of forest musk deer behavior primarily relies on direct human observation or the post hoc playback analysis of ordinary surveillance videos. This approach is not only time-consuming and labor-intensive but also highly subjective, easily leading to missing or misjudged critical behavioral information. Moreover, it is difficult to achieve real-time monitoring and anomaly warning. These limitations severely constrain the efficiency of the large-scale artificial breeding of forest musk deer and the effective advancement of wild population conservation. Thus, this study proposes a forest musk deer behavioral recognition method based on an improved YOLOv8-Pose. A forest musk deer behavior image dataset covering four typical behaviors was constructed, and 18 keypoints were systematically annotated. This study designs a Dilated Spatial Pyramid Pooling-Fast (DILATED-SPPF) module and a Multi-scale Depthwise Separable Context Mixer (MDSC-Mixer) module, and integrates them into YOLOv8-Pose. Experimental results show that the improved model outperforms the original YOLOv8-Pose and comparison models such as YOLOv11/v12-Pose on key metrics of object detection (Box-mAP50 0.929, Box-mAP50-95 0.814) and pose estimation (Pose-mAP50 0.879, Pose-mAP50-95 0.565). This study further develops a visual interactive interface that intuitively presents detection results and skeleton structures. This work provides a high-precision, low-cost automated behavior analysis tool for the artificial breeding and wild conservation of forest musk deer with significant application value for enhancing the intelligence level of endangered species protection.

## Simple Summary

Traditional forest musk deer behavior monitoring relies on manual observation or video playback, which suffers from low efficiency, strong subjectivity, and difficulty in real-time warning, thus restricting breeding and conservation efforts. To address these problems, this study proposes a behavioral recognition method based on an improved YOLOv8-Pose. By constructing a keypoint dataset covering four behaviors and introducing optimized modules, the accuracy of object detection and pose estimation is enhanced, outperforming several existing models. A visual interface is also developed, providing an efficient, low-cost automated analysis tool for the artificial breeding and wild conservation of forest musk deer, thereby contributing to the intelligent protection of endangered species.

## 1. Introduction

Forest musk deer (scientific name: Moschus berezovskii) belong to the genus Moschus, family Moschidae, order Artiodactyla. It is a small, timid forest ungulate. Its most distinctive feature is that males possess a musk gland in the abdomen that secretes musk, a substance of high medicinal and aromatic value. In traditional Chinese medicine, musk is a precious ingredient for resuscitating and activating blood circulation; in the perfume industry, it is an indispensable fixative for many luxury perfume brands. However, due to long-term overhunting and habitat fragmentation, the wild population of forest musk deer has sharply declined. It is now listed as a national first-class protected wild animal in China, making artificial breeding and scientific conservation core measures to ensure the species’ survival.

In the artificial breeding and wild conservation of forest musk deer, behaviors such as feeding, resting, reproduction, and stress responses are core indicators for assessing individual physiological health, environmental adaptability, and reproductive potential, directly affecting the quality of husbandry management and the effectiveness of conservation strategies. Traditional forest musk deer behavior monitoring relies on direct human observation or the post hoc playback of ordinary surveillance videos. This mode not only consumes substantial human and material resources with low efficiency but also suffers from strong subjectivity, easily missing critical behavioral information or causing misjudgments. It is even more difficult to achieve real-time monitoring and abnormal behavior warning, seriously restricting the advancement of the large-scale artificial breeding of forest musk deer and the precision of wild population conservation.

With the rapid development of computer vision and deep learning, keypoint detection technology based on deep learning, with its advantages of non-invasiveness, high precision, and automation [1,2,3,4], has become a core solution to break through the bottlenecks of traditional monitoring. This technology accurately locates key joints of the animal skeleton, such as the head, limbs, and trunk [5], constructs behavioral feature vectors, and captures the essential characteristics of behaviors, effectively avoiding the subjectivity and limitations of human observation. As a core supporting technology for animal behavioral recognition, it can achieve the real-time monitoring and high-precision automatic classification of forest musk deer behaviors, providing precise data support for health assessment, stress intervention in artificial feeding, and even the formulation of wild population conservation strategies. This is of great significance for improving the scientific level of forest musk deer protection and breeding.

Keypoint detection technology has been widely applied in the behavioral recognition and health monitoring of livestock such as dairy cows, pigs, and sheep. In pig farming, Chen et al. [6] proposed the PKL-Track method to achieve piglet tracking and activity measurement through keypoint optimization, providing data support for analyzing piglet behavioral rhythms. In equine body measurement, Su et al. [7] integrated object detection and keypoint detection to achieve the automated measurement of Mongolian horse body parameters. Furthermore, Volkmann et al. [8] applied keypoint detection to injury identification in turkey husbandry, judging health status through abnormal changes in skeletal keypoints, thus expanding the application scenarios of the technology. Jia et al. [9] developed a dairy cow lameness detection system based on keypoint tracking, automatically classifying lameness severity by capturing the spatial positions and movement trajectories of limb joints. Li et al. [10] improved a keypoint detection model monitoring oral keypoint information in dairy cows, constructing a rumination behavior motion curve and enabling the real-time tracking of physiological indicators such as chewing frequency and rumination time. Yang et al. [11] realized the automated body measurement of beef cattle based on keypoint detection and local point cloud clustering. Peng et al. [12] combined improved keypoint detection with unilateral depth imaging to enhance the accuracy and efficiency of cattle body dimension measurement. Yang et al. [13] proposed a one-stage keypoint detection network for the end-to-end deployment of cow body measurement.

In aquatic animal research, Zhao et al. [14] proposed a global detail enhancement and keypoint region fusion method to achieve the fine-grained individual recognition of underwater fish, providing technical support for fish behavior monitoring. Wu et al. [15] realized the computation and analysis of phenotypic parameters of mud crabs (Scylla paramamosain) based on the YOLOv11-DYPF keypoint detection model. Chen et al. [16] improved the YOLOv8 model to achieve carapace keypoint detection and the size measurement of Chinese mitten crabs, with accuracy and real-time performance meeting practical application requirements. In insect behavioral recognition, Sledevič et al. [17] proposed a keypoint-based bee orientation estimation and hive entrance slope detection system, achieving automated bee behavioral recognition through head and thorax keypoint localization, providing a new tool for insect behavioral ecology research.

In recent years, lightweight design, cross-species adaptation, and multimodal fusion have become core trends. Zhao et al. [18] proposed the ALIKE network, achieving 95 FPS real-time detection performance through a differentiable keypoint detection module and lightweight architecture, with excellent performance in tasks such as homography estimation and camera pose estimation. Their subsequent ALIKED network [19] introduced a sparse deformable descriptor head to further reduce computational overhead and improve geometric invariance. To address species diversity, Zhang et al. [20] proposed an open-vocabulary animal keypoint detection method that achieves the keypoint recognition of arbitrary species through semantic feature matching, providing a new idea for cross-species transfer.

The above studies provide key theoretical references and technical support for forest musk deer behavioral recognition. However, forest musk deer behavioral recognition still faces many unique challenges. The living environment of forest musk deer is complex, and their behaviors are significantly influenced by environmental factors such as different light intensities, temperatures, and humidities, which interfere with the accurate capture and analysis of behavioral features. Moreover, some forest musk deer behaviors are transient and sudden, such as instant fleeing when startled, or sudden aggressive acts, which demand high real-time performance and sensitivity from the behavioral recognition system. Additionally, the fur color of forest musk deer is highly similar to the background of captive environments, which can lead to missed detections.

In summary, this study proposes a forest musk deer behavior detection framework based on an improved YOLOv8-Pose model, achieving the high-precision, automated recognition of forest musk deer posture and behavior through deep learning. The framework can monitor and automatically classify typical forest musk deer behaviors (such as lying/sleeping, standing/walking, foraging, and defecation) in real time, and can be effectively applied in artificial breeding and wild conservation scenarios. The system can assist breeders or researchers in the timely detection of abnormal behaviors, assessment of individual health status, and optimization of feeding management strategies, thereby improving forest musk deer welfare and population breeding efficiency. Furthermore, the framework can provide substantial objective, quantifiable posture data for behavioral ecology research, promoting the scientific and refined conservation of forest musk deer.

The DILATED-SPPF and MDSC-Mixer modules proposed in this study enhance the model’s multi-scale feature fusion and contextual understanding capabilities with only a modest increase in computational cost, resulting in improved accuracy in object detection and keypoint localization. This provides scientific decision-making for intelligent wildlife conservation and promotes the deep integration of computer vision with ecological protection disciplines, with significant research significance and application prospects.

## 2. Materials and Methods

This section introduces the improved YOLOv8-Pose model for forest musk deer behavior detection, including model selection, dataset construction, and model improvements.

### 2.1. Framework

**Figure 1:**
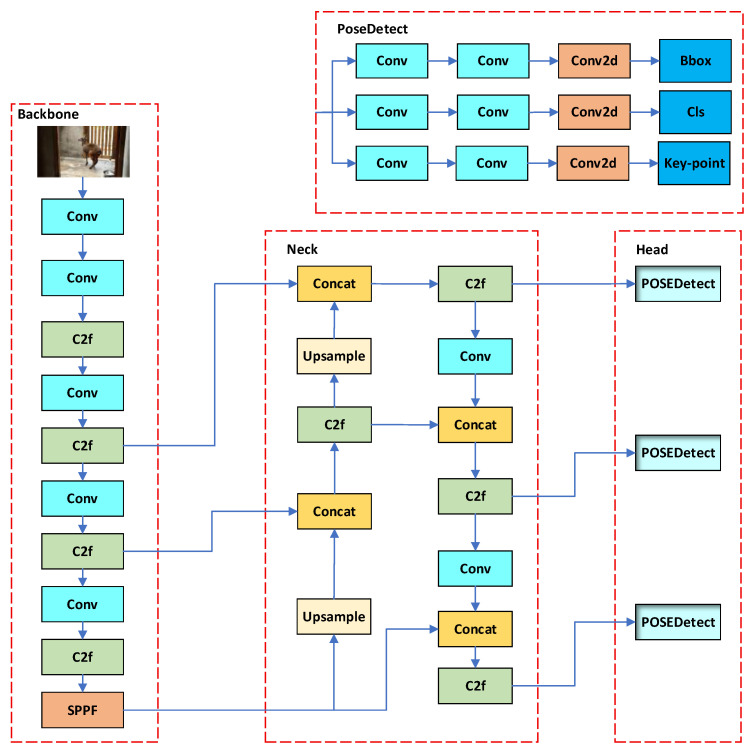
YOLOv8-Pose network structure diagram.

Given the requirements of real-time performance and the accuracy for forest musk deer behavior detection, YOLOv8-Pose was adopted as the keypoint detection algorithm [21,22,23,24]. YOLOv8-Pose adopts an end-to-end integrated design that fuses object detection and keypoint regression into a single network, outputting both individual positions and their keypoint coordinates in one forward pass [25]. This approach offers significant speed advantages while maintaining high accuracy, and is easy to deploy, making it particularly suitable for the real-time monitoring of specific objects such as forest musk deer. Moreover, compared to YOLOv11-Pose and YOLOv12-Pose, YOLOv8-Pose demonstrates a more balanced accuracy between detection boxes and keypoints for forest musk deer behavior detection. Figure 1 shows the network structure of YOLOv8-Pose, including the backbone, neck, and pose head. The backbone uses the CSPDarknet structure to efficiently extract multi-level semantic features through cross-stage partial connections. The neck enhances the spatial representation capability by fusing multi-scale features, combining deep semantic information with shallow high-resolution features. The detection head adopts a decoupled design, outputting bounding boxes, classification, and keypoint coordinates and confidences in parallel, enabling the collaborative output of complete detection and pose estimation results in one forward pass.

**Figure 2:**
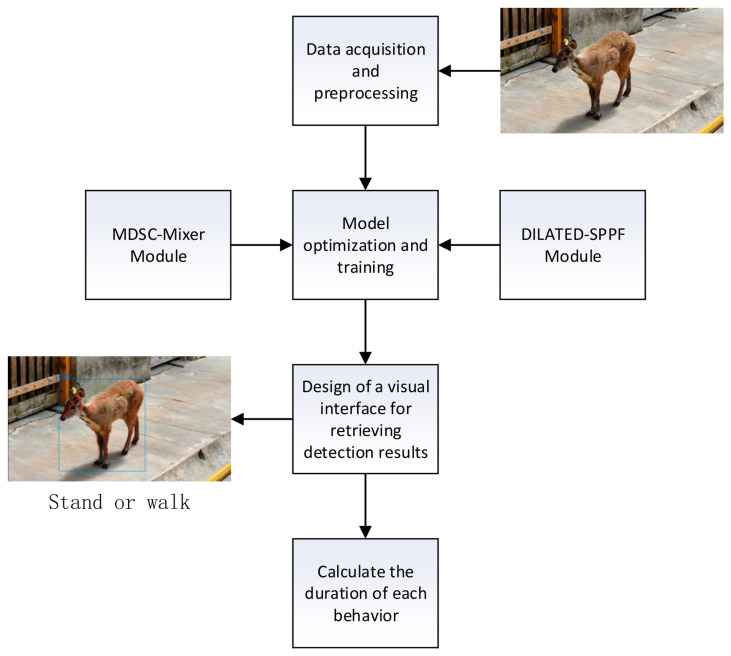
System workflow diagram.

Figure 2 presents the overall system workflow. First, forest musk deer image data are collected and preprocessed, and these raw data are annotated and format-standardized to improve subsequent processing accuracy and efficiency. Subsequently, model optimization and training are carried out. The DILATED-SPPF and MDSC-Mixer modules are integrated, and an enhanced dataset is used to boost model performance, ensuring the effective recognition of various forest musk deer behavior patterns. Finally, a visual interface is designed to present model outputs in a graphical, interactive manner, facilitating user viewing and analysis.

### 2.2. Dataset Construction

#### 2.2.1. Data Collection

**Figure 3:**
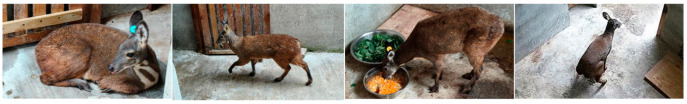
Forest musk deer behaviors.

To ensure model generalization ability and detection accuracy, image data collection and processing were conducted at a provincial forest musk deer breeding base. Data were acquired using mobile shooting devices and fixed-point cameras. Two cameras were deployed in each enclosure to achieve the full coverage of feeding, resting, and activity areas. Data were collected from approximately 15 to 20 healthy adult forest musk deer individuals. Figure 3 shows the collected forest musk deer behaviors, covering four typical behaviors: “lying/sleeping, standing/walking, foraging, and defecation” [26].

#### 2.2.2. Data Annotation

**Figure 4:**
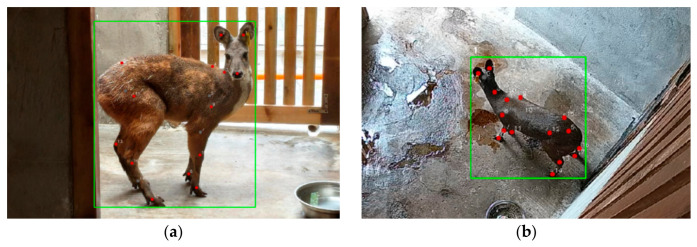
Keypoint data annotation. (**a**) Mobile shooting devices data annotation. (**b**) Fixed-point cameras data annotation. The green box represents the detection box of the forest musk deer, and the numbers 0, 1, 2, 3, in the top left corner represent the musk deer’s behaviors: lying/sleeping, standing/walking, foraging, defecation. The red dots represent visible keypoints, and the gray dots represent invisible keypoints.

Figure 4 shows data annotation from mobile shooting devices and fixed-point cameras. Each image required the precise labeling of 18 core keypoints of the forest musk deer [27,28], including ear tips, nose tip, neck, shoulder, hip, forelimb knee, hindlimb knee, forelimb hoof, hindlimb hoof, upper spine, and lower spine. Table 1 records the annotation positions of the keypoints. All images were annotated by a single annotator following a pre-defined guideline for the 18 keypoints. To minimize errors, the annotator performed multiple rounds of self-checking and re-examined each keypoint placement. After the initial annotation, two senior researchers with expertise in forest musk deer independently reviewed all keypoint positions and visibility flags. Any ambiguous or inconsistent cases were discussed and corrected jointly until a consensus was reached. This process ensured high annotation accuracy and reliability [29,30]. A total of 948 forest musk deer behavior images were annotated and divided into training and validation sets at a ratio of 8:2. Table 2 shows the raw data volumes for different behaviors.

#### 2.2.3. Data Augmentation

In the preprocessing stage, data augmentation operations were applied to enhance data diversity and model robustness: Horizontal flipping: images were mirrored left–right to increase data diversity, simulating the posture characteristics of forest musk deer facing different directions in enclosures. Random rotation: images were rotated within a range of −10° to +10°, with random angles per rotation, and edge padding to maintain image integrity and avoid the loss of key regions. Brightness and contrast adjustment: brightness adjustment range was set to −30 to +30, changing the overall brightness via linear transformation to simulate shooting conditions under different illuminations; contrast adjustment range was 0.8 to 1.2 times, enhancing or weakening image contrast by stretching or compressing pixel value distribution, improving model robustness to lighting variations. Gaussian noise: Gaussian noise was added with mean 0 and standard deviation 25 to simulate random interference during sensor acquisition, enhancing the model anti-noise capability. Combined augmentation: horizontal flipping was first applied with 50% probability, followed by brightness and contrast adjustments added on the flipped images, further enriching data diversity and complexity, enabling the model to learn more comprehensive posture feature representations. These augmentation operations were applied to the divided datasets, resulting in a total of 4740 augmented forest musk deer behavior images, with 3785 in the training set and 955 in the validation set. Table 3 shows the volume of data for different behaviors after applying data augmentation.

### 2.3. Model Improvements

#### 2.3.1. DILATED-SPPF Module Improvement

To improve the detection accuracy of bounding boxes and keypoints, this study designed the DILATED-SPPF module and the MDSC-Mixer module. The DILATED-SPPF module restructures the original serial SPPF [31] pooling structure into a parallel multi-scale pooling structure, using three pooling kernels of different sizes (3 × 3, 5 × 5, 7 × 7) to simultaneously extract multi-level spatial features, thereby improving feature extraction efficiency. Four groups of 3 × 3 dilated convolutions [32] with different dilation rates (1, 2, 3, 4) are introduced to expand the receptive field while maintaining feature map resolution, effectively capturing multi-scale contextual information. The outputs of each branch are weighted fused, and an Efficient Channel Attention (ECA) module is integrated to recalibrate channel weights of the concatenated features, enhancing the expression of key features. This improved module achieves the synergistic optimization of receptive field expansion and multi-scale feature fusion with almost no increase in computational complexity, providing a stronger multi-scale representation capability for object detection in complex scenes [33,34].

**Figure 5:**
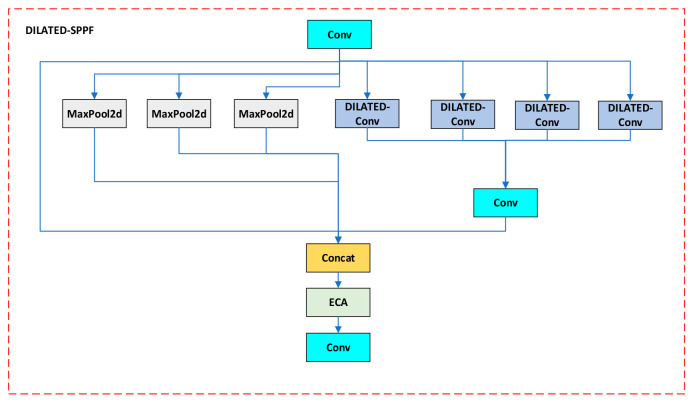
DILATED-SPPF module network structure diagram.

Comparison with existing multi-scale modules: The proposed DILATED-SPPF module differs from conventional multi-scale pooling, dilated convolution, and attention-based feature enhancement methods in several crucial aspects. The original SPPF used in YOLOv8 employs three serial 5 × 5 max-pooling operations, which progressively expand the receptive field but can cause a loss of fine-grained spatial information due to repeated down-sampling-like effects. In contrast, DILATED-SPPF adopts parallel pooling branches with different kernel sizes, simultaneously preserving multi-scale features. Moreover, unlike the Atrous Spatial Pyramid Pooling (ASPP) commonly used in semantic segmentation, which stacks dilated convolutions without pooling, our design combines parallel max-pooling and dilated convolutions and then employs ECA attention for adaptive channel-wise fusion. The four parallel dilated convolutions with rates of 1, 2, 3, and 4 are specifically chosen to capture the size variations of forest musk deer body parts, from small keypoints (e.g., ear tips) to larger structures (e.g., torso), without increasing computational cost. Compared to standard attention modules such as SE or Convolutional Block Attention Module (CBAM), the integration of ECA after multi-source feature concatenation enables efficient cross-channel interaction while remaining lightweight. Thus, DILATED-SPPF is a tailored multi-scale enhancement for single-target animal keypoint detection in cluttered captive environments. Figure 5 shows the network structure of the DILATED-SPPF module.

#### 2.3.2. MDSC-Mixer Module Improvement

MDSC-Mixer is a lightweight context feature enhancement unit specifically designed for pose estimation performance. Its core design goal is to enrich multi-scale contextual information [35] while maintaining low computational and parameter overhead, effectively addressing keypoint detection accuracy challenges in pose estimation tasks.

The module processes the input feature map through three parallel branches. The residual branch, via an optional 1 × 1 convolution for identity mapping or channel alignment, provides a direct gradient pathway to the backbone features, ensuring training stability. The Squeeze-and-Excitation (SE) channel attention branch captures the global statistical information of each channel through global average pooling, then learns inter-channel importance relationships through two fully connected layers, ultimately generating attention weights. This mechanism allows the network to adaptively emphasize feature channels relevant to pose estimation while suppressing irrelevant or noisy channels.

The core innovation of the module lies in its Multi-scale Depthwise Separable Context (MDSC) branch. This branch deploys four depthwise separable convolution units in parallel, each with different dilation rates (1, 2, 3, 4). Each unit first performs dilated depthwise convolution to extract spatial features with different receptive fields, followed by pointwise convolution for cross-channel feature fusion and transformation. The increasing dilation rates enable this branch to systematically capture multi-level spatial context information ranging from local details and part relationships to overall configurations.

The outputs of each branch are integrated through a feature fusion module. The multi-scale features are first concatenated along the channel dimension, then element-wise multiplied with the channel attention weights to achieve the weighted fusion of context information. The fused features are then passed through a 1 × 1 convolutional layer for dimensionality reduction and non-linear activation, and finally summed with the output of the residual branch to complete the residual connection. The entire processing flow maintains the spatial resolution of the input and output feature maps unchanged, ensuring their seamless integration into existing network architectures.

**Figure 6:**
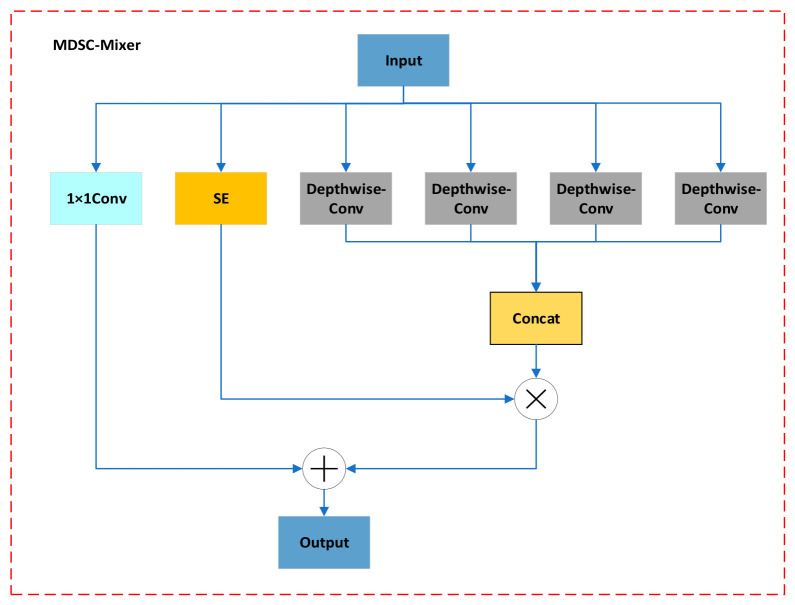
MDSC-Mixer module network structure diagram.

Differentiation from existing attention and context modules for pose estimation: The MDSC-Mixer specifically addresses the limitations of conventional attention mechanisms and multi-scale fusion strategies when applied to animal pose estimation. Standard channel attention modules like SE and spatial-channel modules like CBAM recalibrate features based on single-scale representations and lack the ability to explicitly model spatial relationships across different ranges. While dilated convolution-based designs such as ASPP or DenseASPP capture a multi-scale context, they typically rely on standard convolutions, which lead to a heavy parameter footprint, and do not simultaneously integrate channel attention and residual learning for keypoint refinement. In contrast, the MDSC-Mixer employs parallel depthwise separable dilated convolutions for lightweight multi-scale context aggregation, while a dedicated SE branch models global inter-channel dependencies. The element-wise multiplication between the multi-scale context features and the channel attention weights enables the network to emphasize the most informative scale for each keypoint. The residual connection further ensures the preservation of original backbone features, which is crucial for training stability. Compared to recent pose estimation approaches that rely on high-resolution feature maps or iterative refinement, the MDSC-Mixer is a compact plug-in module that enhances contextual understanding without altering feature map resolution, making it particularly suitable for the real-time keypoint detection of forest musk deer. This joint optimization of multi-scale spatial encoding, channel attention, and feature preservation in a single lightweight unit is the core distinction of our design. Figure 6 shows the network structure of the MDSC-Mixer module.

#### 2.3.3. Improved YOLOv8-Pose Network Structure

**Figure 7:**
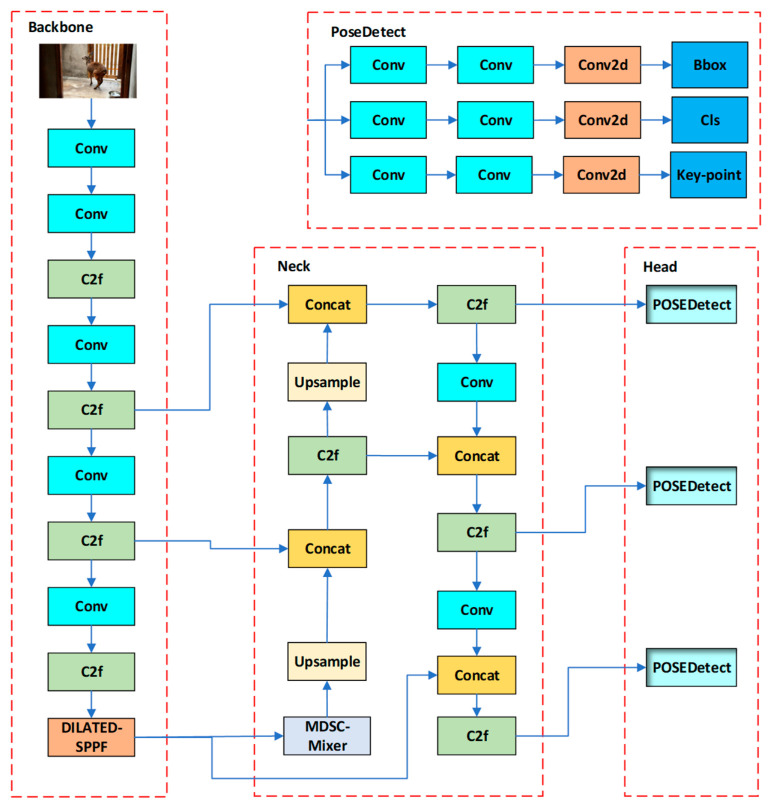
Improved YOLOv8-Pose network structure diagram.

The two improved modules are integrated into the YOLOv8-Pose framework, as illustrated in Figure 7. In the designed architecture, the MDSC-Mixer module is placed in the neck network, while the DILATED-SPPF layer is positioned at the end of the backbone. Through this structural arrangement, the two modules jointly establish an optimization chain of “context enhancement–multi-scale receptive field expansion–key feature strengthening” across the backbone–neck junction. The MDSC-Mixer enriches the feature representations with multi-scale contextual information within the neck, which is then synergistically combined with the dilated spatial pyramid pooling in the backbone. Consequently, the network acquires stronger multi-scale representation capabilities in complex scenes, leading to improved detection accuracy and enhanced localization robustness for pose estimation tasks. Subsequent additional experiments further verify the rationality of this design.

### 2.4. Evaluation Metrics

#### 2.4.1. Object Detection Evaluation Metrics

In the YOLOv8-Pose model, the Precision metric for detection boxes is used to evaluate the localization accuracy of object detection boxes at a given confidence threshold. Since YOLOv8-Pose performs both object detection and keypoint regression simultaneously, detection box Precision is a fundamental metric to measure whether the model “correctly locates the object’s position”. To evaluate the accuracy of detection boxes, the Intersection over Union (*IoU*) between the predicted box and the ground truth box is first defined.

For any predicted bounding box $B_{p}$ and ground truth bounding box $B_{gt}$, *IoU* is defined as the ratio of the area of their intersection to the area of their union, as shown in Formula (1):

(1) $$IoU=\frac{B_{p}⋂B_{gt}}{B_{p}⋃B_{gt}}$$

where ⋂ and ⋃ represent the areas of intersection and union, respectively. *IoU* ranges from [0, 1]; the closer its value to 1, the higher the spatial overlap between the predicted box and the ground truth box. During evaluation, an *IoU* threshold θ is typically set; when IoU≥θ, the prediction is considered a true positive (*TP*), otherwise it is considered a false positive (*FP*).

Precision (*P*) measures the proportion of true positive predictions among all positive predictions made by the model, and is defined as the ratio of TP to the sum of TP and FP, as shown in Formula (2):

(2) $$P=\frac{TP}{TP+FP}$$

where TP is the number of true positives whose *IoU* with the ground truth exceeds the threshold, and FP is the number of false positives.

Recall (*R*) is the proportion of actual positive samples correctly predicted as positive by the model, as shown in Formula (3):

(3) $$R=\frac{TP}{TP+FN}$$

where FN (False Negative) represents actual targets not detected by the model.

To comprehensively evaluate the overall performance of precision and recall, Average Precision (*AP*) is introduced. AP is defined as the area under the Precision–Recall curve, which plots precision against recall, as shown in Formula (4):

(4) $$AP=\int_{0}^{1}P(R)dR$$

For detection tasks with multiple categories, the mean Average Precision (*mAP*) is used to aggregate the AP across all categories, as shown in Formula (5):

(5) $$mAP=\frac{1}{C}\sum_{i=1}^{C}{AP}_{i}$$

where C is the total number of categories.

To evaluate model performance under different localization accuracy requirements, *mAP* is typically computed at multiple *IoU* thresholds, resulting in the following two common metrics: *mAP50*: *mAP* computed at an *IoU* threshold of 0.5. This metric has higher tolerance for localization errors and is mainly used to measure the model’s ability to detect objects. *mAP50-95*: The average of *mAP* computed at *IoU* thresholds from 0.5 to 0.95 with a step size of 0.05 (10 thresholds in total). This metric imposes extremely strict requirements on localization accuracy and comprehensively reflects model performance in high-precision localization scenarios.

#### 2.4.2. Keypoint Evaluation Metrics

In pose estimation, in addition to detection boxes, the prediction quality of keypoints must be evaluated. Since the number of keypoints is fixed and spatially correlated, traditional *IoU*-based metrics are not applicable; therefore, Object Keypoint Similarity (*OKS*) is introduced as the fundamental measure.

For a single detection instance, the *OKS* value measures the overall similarity between the predicted keypoint set and the ground truth keypoint set, as shown in Formula (6):

(6) $$OKS=\frac{\sum_{i}exp(-\frac{d_{i}^{2}}{2s^{2}k_{i}^{2}})\cdot \delta (v_{i}>0)}{\sum_{i}\delta (v_{i}>0)}$$

where $d_{i}$ is the Euclidean distance between the predicted position and the ground truth position of the i-th keypoint; s is the scale of the target in the image, typically the square root of the detection box area; $k_{i}$ is a normalization factor for the i-th keypoint, used to adjust the tolerance for different types of keypoints (e.g., trunk keypoints have higher tolerance than limb extremities); $v_{i}$ is the visibility flag of the keypoint, and $\delta (v_{i}>0)$ includes only visible or annotated keypoints. *OKS* ranges from [0, 1]; the closer its value to 1, the more consistent the predicted keypoint positions are with the ground truth. This metric combines spatial distance normalization and keypoint-specific weighting, allowing the fair evaluation of localization accuracy for targets of different scales and different body parts.

After obtaining the *OKS* value for each predicted keypoint, analogous to the *IoU*-based evaluation in object detection, when *OKS* is greater than or equal to a threshold *T*, the keypoint prediction is considered correct. Precision is defined as in Formula (7):

(7) $$P_{K}=\frac{{TP}_{K}}{{TP}_{K}+{FP}_{K}}$$

where ${TP}_{K}$ is the number of true positives with OKS≥T, and ${FP}_{K}$ is the number of false positives.

Recall ($R_{K}$) is the proportion of actual positive samples correctly predicted as positive by the model, as shown in Formula (8):

(8) $$R_{K}=\frac{{TP}_{K}}{{TP}_{K}+{FN}_{K}}$$

where ${FN}_{K}$ (False Negative) represents actual keypoints not detected.

Similar to detection boxes, ${AP}_{K}$ for keypoints is defined as the area under the Precision–Recall curve, as shown in Formula (9):

(9) $${AP}_{K}=\int_{0}^{1}P_{K}(R_{K})dR_{K}$$

The mean Average Precision (${mAP}_{K}$) aggregates ${AP}_{K}$ across all keypoints, as shown in Formula (10):

(10) $${mAP}_{K}=\frac{1}{K}\sum_{i=1}^{K}{{AP}_{K}}_{i}$$

where K is the total number of keypoints.

Keypoint ${mAP}_{K}$*50* is the mean of ${AP}_{K}$ over all keypoint categories at a fixed *OKS* threshold of 0.5. It is a core intuitive metric in pose estimation, representing keypoint detection performance under relaxed matching conditions. Keypoint ${mAP}_{K}$*50-95* is the average of keypoint ${mAP}_{K}$ computed at *OKS* thresholds from 0.5 to 0.95 (step 0.05, 10 thresholds). It comprehensively characterizes the robustness of keypoint localization under varying strictness levels.

## 3. Experiments and Results

The hardware and software systems and parameters used in this experiment are as follows: Windows 11 operating system, NVIDIA GeForce RTX 5090 GPU, Intel(R) Core(TM) Ultra 9 285K, torch-2.8.0 framework, and Python-3.12.11, CUDA-12.8.

### 3.1. Comparison Experiments

As shown in Table 4, our model exhibits strong overall performance in the detection and pose estimation of captive forest musk deer. All frame rate (FPS) measurements were conducted on the same video clip of captive forest musk deer without drawing skeleton connections on the visualization interface, ensuring a fair and consistent comparison of raw model performance (in actual deployment with visualization enabled, the frame rate will be lower). Keypoint R-CNN is a classic two-stage pose estimator built upon Mask R-CNN that predicts keypoints for each detected instance, but its heavy backbone leads to high computational cost. For Keypoint R-CNN, its GFLOPs cannot be properly computed (marked as “——” in the table) due to its complex two-stage architecture and non-standard operators. Moreover, its large parameter count (59.2 M) and low FPS (25.59) make it impractical for real-time detection. Therefore, Keypoint R-CNN is excluded from the main comparison, and subsequent analysis focuses on lightweight models that meet the real-time requirements.

Under this constrained setting, our improved model demonstrates clear advantages. In object detection, its Box-mAP50 and Box-mAP50-95 reach 0.929 and 0.814, respectively, outperforming all compared YOLO-based pose models, indicating improved localization accuracy for captive forest musk deer. In pose estimation, it achieves a Pose-mAP50 of 0.879 and a Pose-mAP50-95 of 0.565, which are higher than those of YOLOv8n-Pose, YOLOv11n-Pose, and YOLOv12n-Pose, and slightly lower than YOLO26n-Pose (0.884 and 0.594). Meanwhile, the model maintains a moderate footprint with 3.66 M parameters, 9.6 GFLOPs, and an inference speed of 90.61 FPS, striking a favorable balance between accuracy and efficiency that satisfies the demands of real-time detection.

Nevertheless, certain limitations remain. Compared with extremely lightweight variants such as YOLOv11n-Pose and YOLOv12n-Pose, our model exhibits a slightly higher parameter count and computational cost. Its inference speed is lower than that of YOLOv8n-Pose (104.5 FPS) and YOLO26n-Pose (95.46 FPS). Moreover, the model still slightly lags behind YOLO26n-Pose on the pose estimation metrics (Pose-mAP50 and Pose-mAP50-95), suggesting room for further improvement in keypoint regression accuracy.

### 3.2. Ablation Experiments

The results of the ablation study are presented in Table 5. Starting from the YOLOv8n-Pose baseline (Box-P 0.893, Box-mAP50 0.925, Pose-P 0.875, Pose-mAP50 0.854, 3.312M parameters, 9.3 GFLOPs), we evaluate the individual and combined effects of the DILATED-SPPF and MDSC-Mixer modules. Adding DILATED-SPPF alone improves the Box-P to 0.915 and the Pose-mAP50 to 0.868, while the Pose-P remains unchanged at 0.875, suggesting that this module enhances spatial feature representation and keypoint recall without sacrificing precision. Introducing a MDSC-Mixer alone yields a Box-P of 0.926 and a Box-mAP50 of 0.932, together with a Pose-P of 0.881, demonstrating its strength in fine-grained localization; however, Pose-mAP50 (0.864) shows only a modest gain over the baseline. When both modules are integrated, the model achieves the best pose estimation performance, with Pose-P reaching 0.897 and Pose-mAP50 reaching 0.879, reflecting a clear synergistic effect on keypoint accuracy. For object detection, the combined model attains a Box-P of 0.906 and a Box-mAP50 of 0.929, which are slightly lower than the best single-module results but still markedly outperform the baseline. The overall parameter count and computational cost increase slightly to 3.66 M and 9.6 GFLOPs, respectively. In summary, both DILATED-SPPF and MDSC-Mixer bring improvements over the baseline. The combined model achieves the highest pose estimation accuracy; however, its detection metrics (Box-P and Box-mAP50) are slightly inferior to those obtained with the MDSC-Mixer alone, indicating a minor trade-off between detection and pose estimation performance.

### 3.3. Additional Experiment

**Figure 8:**
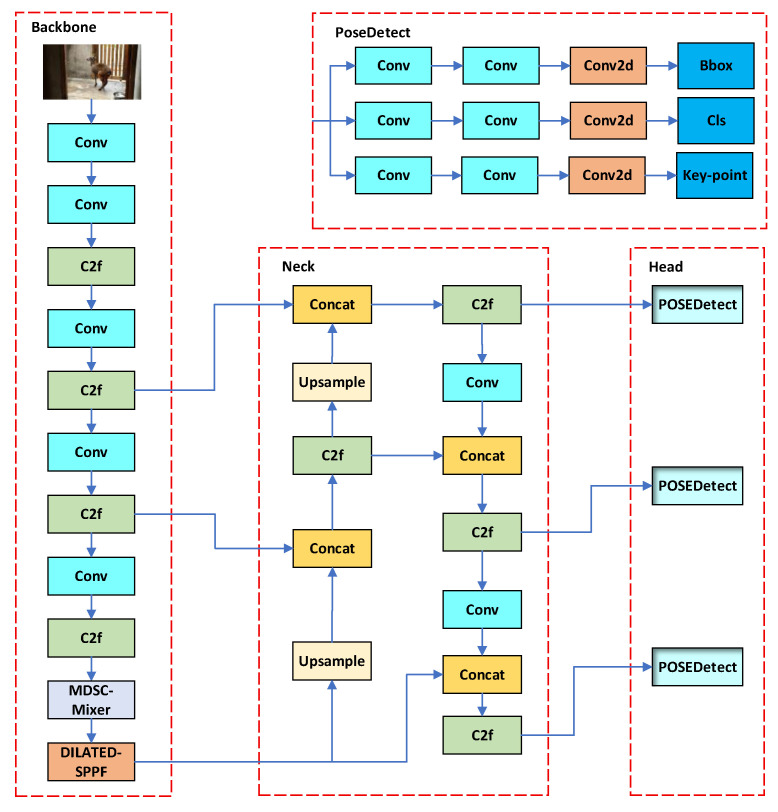
Network architecture diagram of Experiment 1.

**Figure 9:**
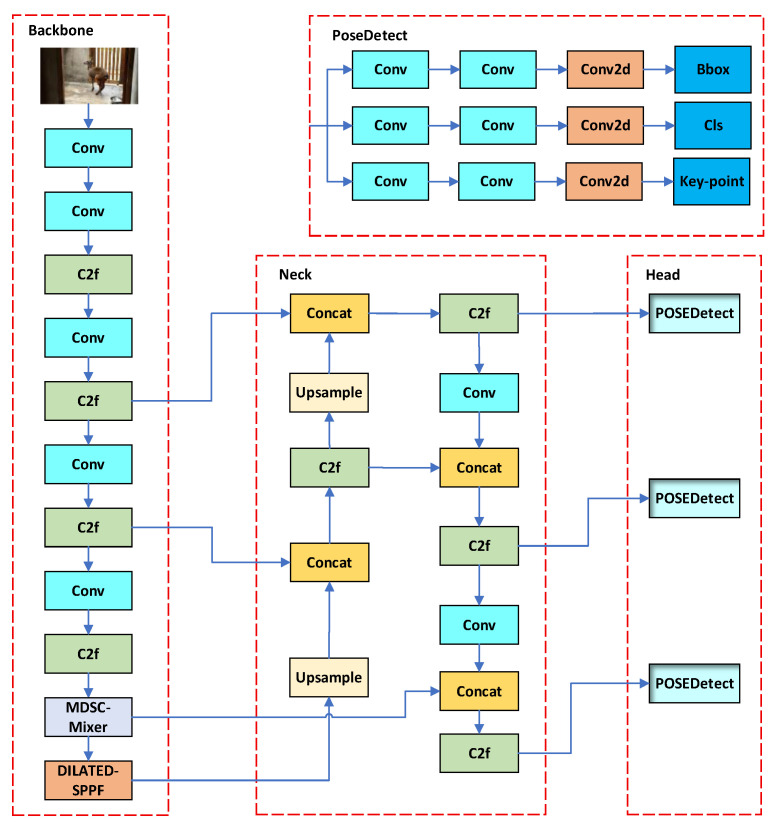
Network architecture diagram of Experiment 2.

**Figure 10:**
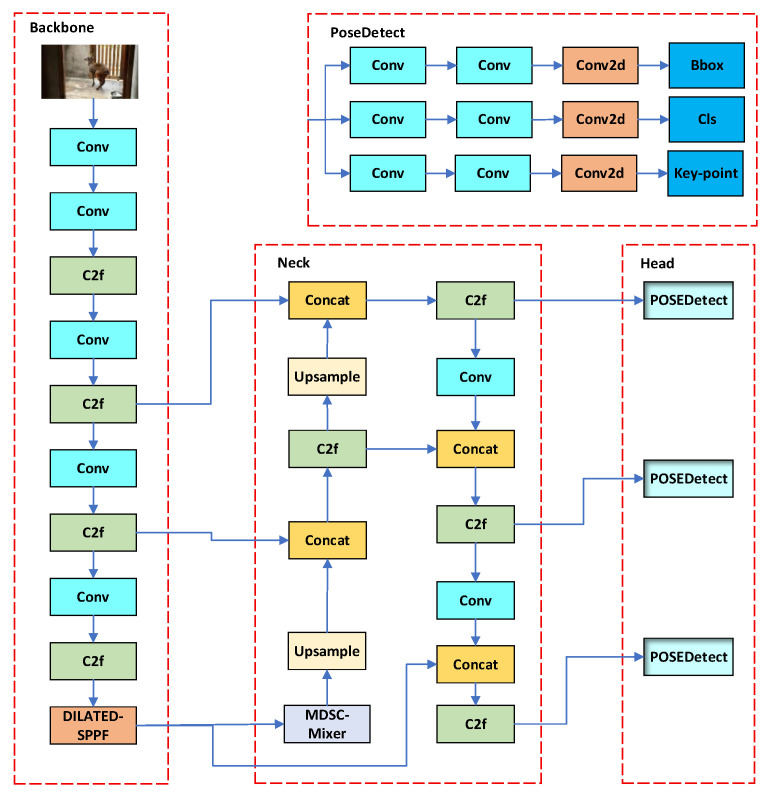
Network architecture diagram of Experiment 3.

**Figure 11:**
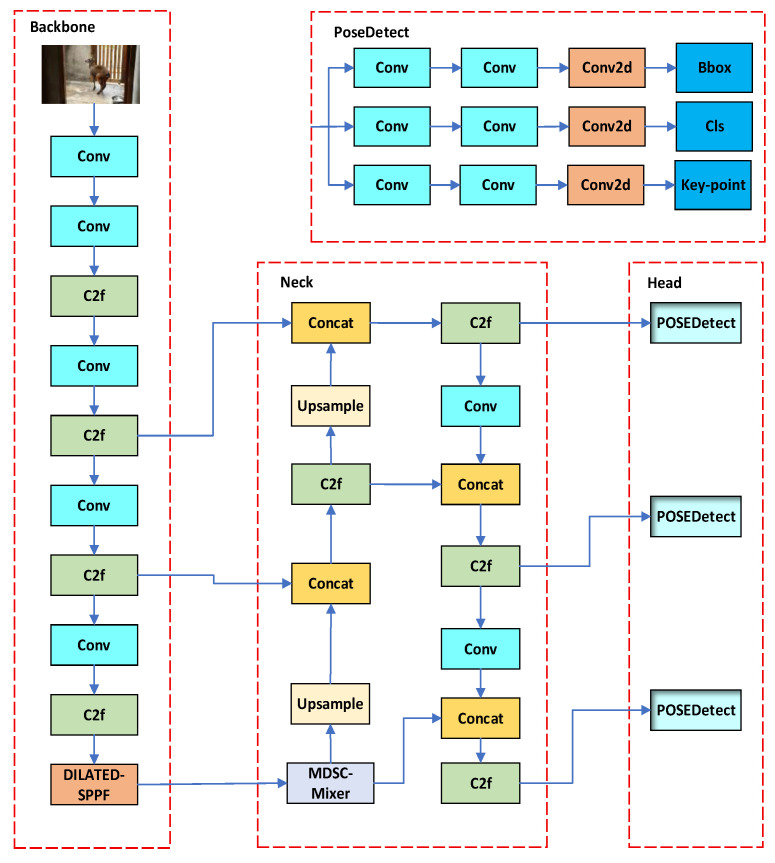
Network architecture diagram of Experiment 4.

Through four sets of additional experiments in Table 6, this paper systematically investigates the influence of the placement of the MDSC-Mixer module and the network connection structure on both object detection and pose estimation performance. The six metrics obtained from Experiment 1 and Experiment 4 are completely identical, indicating that even when the positions of MDSC-Mixer and DILATED-SPPF are exchanged between the backbone and neck networks without altering the network connection structure, the representational capacity and convergence results of the model remain equivalent. This demonstrates that the rationality of the network connection paths is critical. In Experiment 2, where only the connection structure was adjusted while keeping MDSC-Mixer in the backbone, the precision of the pose estimation improved slightly (Pose-P increased from 0.882 to 0.890), whereas the mean average precision for object detection (Box-mAP50 decreased from 0.925 to 0.923) and for pose estimation (Pose-mAP50 decreased from 0.869 to 0.858, Pose-mAP50-95 decreased from 0.554 to 0.550) both deteriorated. This suggests that inappropriate connection modifications disrupt the information flow between the backbone and neck, impairing the model’s recall and localization consistency for hard samples. Experiment 3, which relocated the MDSC-Mixer to the neck network while maintaining a reasonable connection, achieved the most outstanding overall performance: the object detection accuracy (Box-mAP50 reached 0.929, Box-mAP50-95 reached 0.814) and pose estimation accuracy (Pose-P reached 0.897, Pose-mAP50 reached 0.879, Pose-mAP50-95 reached 0.564) all rose to the highest values among the four experiments, with only the Box-P exhibiting a slight decrease from 0.916 to 0.905. This phenomenon can be attributed to the natural trade-off between precision and recall introduced by recalling more hard samples, fully demonstrating that the neck is the ideal location for this module to leverage its advantages in a multi-scale feature fusion, enabling a better balance between precision and recall. In Experiment 4, while moving the module into the neck, the connection structure was concurrently modified, which caused all metrics to regress to the same level as those of Experiment 1 (Box-P 0.916, Box-mAP50 0.925, Box-mAP50-95 0.802, Pose-P 0.882, Pose-mAP50 0.869, Pose-mAP50-95 0.554). The performance gain brought by the neck position was completely offset by the inappropriate connection adjustment. Overall, deploying the MDSC-Mixer in the neck network with a purposefully designed connection structure can maximally enhance the comprehensive accuracy of object detection and human keypoint regression. Moreover, network optimization must treat module placement and information pathways as a unified whole, as any arbitrary modification that deviates from topological equivalence may weaken or even reverse the performance benefits introduced by the module. Network architecture diagrams of Experiments 1–4 are shown in Figure 8, Figure 9, Figure 10 and Figure 11.

### 3.4. Visualization Interface

**Figure 12:**
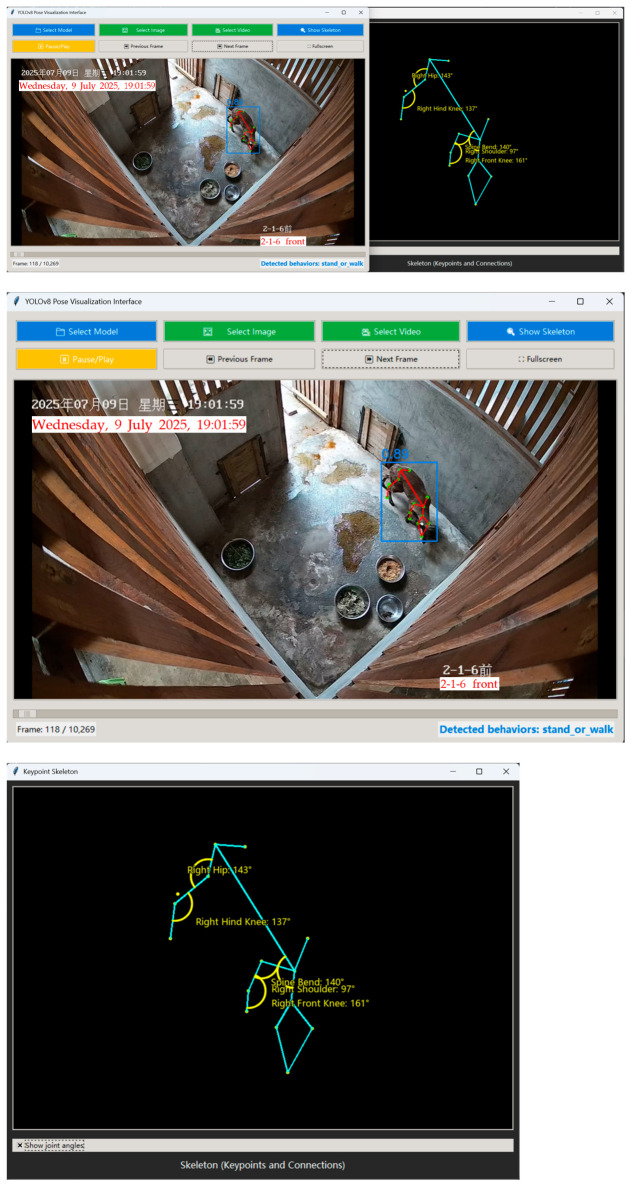
Visual interface display.

In this study, a visual interface was designed for the trained .pt model file, presenting model outputs in a graphical, interactive manner for intuitive user viewing and analysis. Subsequently, keypoints of forest musk deer behaviors were extracted from the recognition results and connected into a complete skeleton model according to the biological structure. Figure 12 shows the visual interface, which includes the following functions: selecting the trained .pt file; choosing image or video detection; optionally displaying the skeleton alone; choosing whether to display the joint angle; recording the duration of each behavior. The designed visual interface enables the real-time detection of forest musk deer behavior and skeleton models, providing a high-precision, low-cost automated behavior analysis tool for the artificial breeding and wild conservation of forest musk deer. Note: Owing to the indelible camera-imposed watermark, the overlaid annotations cannot be removed. The top left corner of the image displays the timestamp Wednesday, 9 July 2025, 19:01:59. The bottom right corner bears the label “2-1-6 front”, which denotes the front camera of sub-enclosure No. 6 within large enclosure 2-1 in the captive forest musk deer facility.

## 4. Discussion

This section discusses the proposed forest musk deer behavioral recognition method, focusing on the effectiveness of the improved modules, the limitations of the dataset and detection challenges in complex scenes, the potential application value of the method, and future research directions.

### 4.1. Impact of Different Improvement Modules on Model Performance

In terms of model improvement, this study designed two modules, namely DILATED-SPPF and MDSC-Mixer, and integrated them into the YOLOv8-Pose network. The ablation experiment results (Table 5) show that after introducing the DILATED-SPPF module alone, the object detection precision (Box-P) increased from 0.893 to 0.915, and the mean average precision for the pose estimation (Pose-mAP50) increased from 0.854 to 0.868, indicating that this module expands the receptive field through parallel multi-scale pooling and dilated convolutions, effectively enhancing the model’s ability to extract features of forest musk deer with different body shapes and scales. After introducing the MDSC-Mixer module alone, the Box-P further increased to 0.926 and the Box-mAP50 reached 0.932, while the keypoint precision (Pose-P) increased from 0.875 to 0.881, verifying the advantage of multi-scale depthwise separable convolutions in capturing contextual information from local details to global configurations. When the two modules were used together, the model achieved the best pose estimation performance (Pose-P 0.897, Pose-mAP50 0.879), demonstrating a clear synergistic enhancement effect on keypoint accuracy. Meanwhile, the combined model attained a Box-P of 0.906 and a Box-mAP50 of 0.929, which are slightly lower than the best single-module results but still markedly outperform the baseline. As shown in Table 4, compared with mainstream models such as YOLOv11-Pose, YOLOv12-Pose, and YOLO26-Pose, the proposed model exhibits superior or comparable performance in both bounding box and keypoint localization, proving the effectiveness and advancement of the proposed improvement strategy for forest musk deer behavioral recognition.

Limitations in strict keypoint localization: Although the proposed model achieves the highest detection precision (Box-mAP50-95 0.814, Table 4) and the best keypoint precision (Pose-P 0.897, Table 5), its Pose-mAP50 (0.879) and Pose-mAP50-95 (0.565) are lower than those of YOLO26n-Pose (0.884 and 0.594, respectively). This discrepancy suggests a trade-off introduced by our lightweight design: The DILATED-SPPF and MDSC-Mixer modules enhance the multi-scale context and global attention, which benefit detection and coarse-level keypoint presence, but the use of depthwise separable convolutions and parallel pooling may lead to subtle spatial detail loss that is critical for precise keypoint coordinate regression under strict OKS thresholds. YOLO26n-Pose likely employs higher-resolution feature maps or a different fusion architecture that better preserves fine-grained spatial information. For forest musk deer monitoring, this limitation means that while the model reliably identifies behaviors and overall pose structure, the exact placement of small keypoints (e.g., ear tips, hoofs) may be less accurate, which could affect very fine-grained behavioral analysis. Future work will explore integrating a lightweight high-resolution feature stream or a keypoint refinement head to mitigate this gap while retaining real-time efficiency.

### 4.2. Limitations of Dataset Scale and Behavior Category Coverage

The forest musk deer behavior image dataset constructed in this study contains 948 original images, which were augmented to 4740 images, covering four typical behaviors: lying or sleeping, standing or walking, foraging, and defecation. However, the dataset still has notable limitations in sample size and behavioral diversity. With only 948 real images, even after augmentation it remains a small-scale dataset, posing a risk of overfitting: the model may inadvertently memorize limited background textures, lighting conditions, and camera angles, leading to an unpredictable degradation in generalization when applied to unseen field scenarios. Additionally, forest musk deer may exhibit complex behaviors in both captive and wild settings, such as grooming, vigilance, fighting, and mother–infant interaction that are not represented in the current dataset, thereby constraining the model’s ability to recognize a broader range of behavioral patterns. Furthermore, data collection was conducted primarily at a provincial forest musk deer breeding base, and the effects of different rearing environments, lighting conditions, seasonal changes, and individual coat color variations on model robustness have not been systematically evaluated. Therefore, the current dataset is representative of captive forest musk deer under the specific management and environmental conditions of a single breeding base, but it does not yet capture the full variability encountered in wild habitats, different farms, or across seasons. This limits the direct generalization of the trained model to broader real-world scenarios. The current diversity also falls short of capturing the full complexity of real-world conditions, including extreme weather, long-distance low-resolution views, partial occlusions, or co-occurrence with similar species.

A further limitation of the present supervised learning approach is its dependence on a relatively large amount of labeled source data, which may restrict scalability and generalizability to new environments, camera settings, or unseen animal populations. To address this, future research could incorporate domain adaptation (DA) techniques to reduce distribution shifts across different visual domains and lessen the need for extensive relabeling. Recent studies have demonstrated the effectiveness of DA in related tasks: Wang [37] employed transformer-based DA for classifying exterior cladding materials in street view images across different cities, and Wang [38] integrated DA with Faster R-CNN for cross-domain non-PPE detection on construction sites. Inspired by these works, in future work we will expand the dataset by incorporating additional behavior categories, more individuals, and images from diverse scenarios—particularly unlabeled surveillance footage from different geographic regions and time periods—and will explore both semi-supervised learning and domain adaptation techniques to leverage such unlabeled data, thereby reducing reliance on costly manual annotation and fundamentally enhancing the model’s adaptability and robustness in practical conservation applications.

### 4.3. Detection Challenges in Complex Scenes

Although the improved model achieved strong overall detection accuracy on the test set, several challenges remain evident in practical application scenarios. The first and most prominent challenge is the natural camouflage of forest musk deer: their grayish-brown coat color is highly similar to the concrete flooring, soil, and withered grass common in captive backgrounds. Under sufficient illumination, texture and contour cues are usually sufficient for the model to distinguish the animal from the background. However, when ambient light is dim—as is frequently the case during dawn, dusk, or in poorly lit corners of enclosures—the contrast between the animal and the background is substantially reduced, and the boundary between target and background becomes ambiguous.

**Figure 13:**
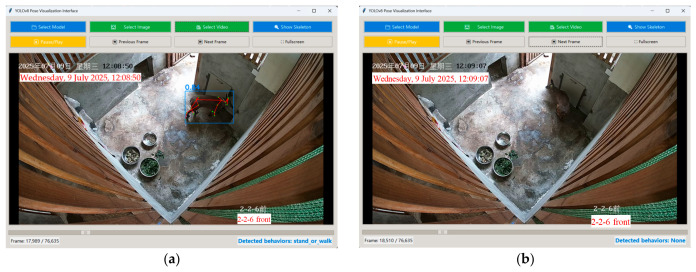
Detection under low-light conditions. (**a**) Successful detection. (**b**) Detection failure.

We illustrate this limitation through representative failure cases captured from surveillance footage. Figure 13a shows a case where normal detection is still achieved under low-light conditions, while Figure 13b shows a case where detection fails under similarly dim illumination. In the failure case, the weak local features in the shadowed regions are insufficient for the pose estimator to establish reliable body part correspondences, leading to missed detections. Notably, although we explicitly applied data augmentation through random adjustments of image brightness and contrast during dataset construction, detection failures under low-light conditions still occurred. This indicates that generic photometric augmentation, while helpful for improving general robustness, is not sufficient to overcome the severe domain gap caused by significantly reduced illumination combined with the natural camouflage of forest musk deer against concrete backgrounds. A more targeted future direction could involve specialized illumination-invariant preprocessing modules or advanced low-light simulation techniques. Additionally, incorporating multimodal sensor data, such as thermal infrared, may offer complementary cues to compensate for the loss of visual information in poor lighting.

A second challenge relates to the transient and sudden nature of certain behaviors. Forest musk deer are timid by nature and may quickly flee or exhibit sudden aggressive movements when startled. The current model performs detection based on single frames and does not yet fully exploit temporal information to capture behavioral dynamics. Consequently, recognizing these instantaneous behaviors remains difficult. Future work may consider introducing lightweight temporal modeling modules, such as optical flow features or short-term memory networks, to improve recognition capability for these rapid behavioral events. Note: Owing to the indelible camera-imposed watermark, the overlaid annotations cannot be removed. The top left corner of the image displays the timestamp Wednesday, 9 July 2025, 12:08:50 and 12:09:07. The bottom right corner bears the label “2-2-6 front”, which denotes the front camera of sub-enclosure No. 6 within large enclosure 2-2 in the captive forest musk deer facility.

### 4.4. Handling of Occlusion in Single-Target Scenes

**Figure 14:**
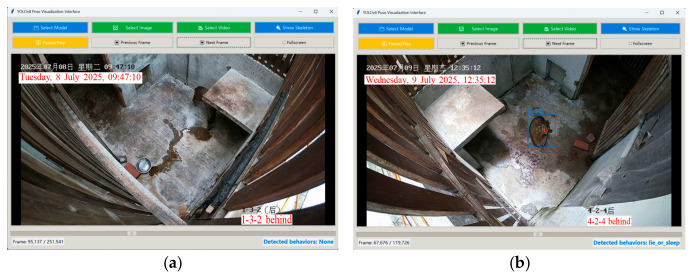
Example of detection failure caused by occlusion. (**a**) Detection failure caused by occlusion from a concrete platform. (**b**) Inaccurate keypoint detection caused by self-occlusion during curling up.

In captive breeding scenarios, forest musk deer are typically housed individually, i.e., one musk deer per enclosure. Therefore, the detection framework in this study is primarily designed for single-target scenes and does not need to address inter-individual occlusion or identity association among multiple animals. Nevertheless, occlusion problems still exist, mainly in two aspects. First, the head, torso, or limbs of the musk deer may be partially occluded by fixed facilities in the enclosure, such as food basins, water basins, or concrete platforms. Figure 14a illustrates a representative case where occlusion by a concrete platform leads to detection failure, as the blocked body regions prevent the model from extracting sufficient features for reliable localization. Second, when the musk deer lies down, curls up, or grooms itself, keypoints on the contralateral limbs or ear tips may be occluded by its own body. As shown in Figure 14b, such self-occlusion while curled up causes inaccurate keypoint detection, reducing the completeness of pose estimation. To address these issues, this study explicitly distinguished the visibility status of keypoints during the data annotation stage, enabling the model to output reasonable confidence rather than forced localization when keypoints are not visible. On this basis, future research can further optimize in the following ways: deploying multi-angle cameras (e.g., front and side-upper views) in the enclosure to improve the overall observability of keypoints through multi-view information fusion, and introducing temporal keypoint interpolation and smoothing methods to reasonably estimate positions during short occlusion periods using motion trajectories from adjacent frames. These improvements will further enhance the robustness and practical value of the proposed method in real captive environments. Note: Owing to the indelible camera-imposed watermark, the overlaid annotations cannot be removed. The top left corner of the image displays the timestamp Tuesday, 8 July 2025, 09:47:10 and Wednesday, 9 July 2025, 12:35:12. The bottom right corner bears the label “1-3-2 behind”, which denotes the behind camera of sub-enclosure No. 2 within large enclosures 1-3 in the captive forest musk deer facility. The label “4-2-4 behind” denotes the behind camera of sub-enclosure No. 4 within large enclosure 4-2 in the captive forest musk deer facility.

### 4.5. Application Value of the Visual Interface and Future Deployment Prospects

The visual interactive interface developed in this study is capable of displaying the detection bounding boxes, keypoints, and the connected skeletal model of forest musk deer in real time. It supports image or video detection, and thereby provides an intuitive and convenient analysis tool for breeders and researchers. In captive breeding scenarios, the interface can assist in monitoring individual health status and promptly detecting abnormal behaviors (e.g., prolonged lack of feeding, frequent defecation, abnormal lying), thereby optimizing feeding management strategies. In wild conservation scenarios, the framework is expected to be deployed on infrared cameras or fixed monitoring points to achieve the long-term, non-invasive behavior monitoring of forest musk deer populations, providing scientific data support for endangered species protection.

However, the computational complexity and model size of the current model (based on YOLOv8n-Pose with a slight increase in parameters and computational load) limit its direct deployment on low-power edge devices (e.g., embedded systems in wild environments). Future research will focus on model lightweighting, adopting techniques such as channel pruning, knowledge distillation, or more efficient convolution modules (e.g., further optimization of depthwise separable convolutions) to reduce computational costs while maintaining detection accuracy. Additionally, integrating edge computing and wireless transmission technologies to construct an end-edge-cloud collaborative intelligent monitoring system will facilitate all-weather, automated behavioral recognition and analysis of forest musk deer, promoting the intelligent development of endangered species conservation.

## 5. Conclusions

This study introduced an automatic behavioral recognition framework for forest musk deer based on an improved YOLOv8-Pose, addressing the critical challenges of traditional manual monitoring, such as low efficiency and strong subjectivity. To overcome the specific technical difficulties posed by the species’ natural camouflage against captive backgrounds and the high variability of its non-rigid postures, we constructed the dedicated keypoint dataset for this endangered species, encompassing four typical behaviors annotated with 18 skeletal keypoints.

Two lightweight modules, DILATED-SPPF and MDSC-Mixer, were designed and synergistically integrated into the YOLOv8-Pose architecture. Rather than isolated enhancements, these modules jointly establish a “spatial multi-scale feature extraction–context-aware feature refinement” optimization pathway. The DILATED-SPPF module expands the receptive field through a parallel architecture of multi-scale pooling and dilated convolutions, while the MDSC-Mixer strengthens fine-grained contextual understanding and keypoint localization via a lightweight, multi-branch design. Beyond merely proposing these modules, our additional experiments rigorously investigated the rationality of their placement and connection within the network, demonstrating that the architecture itself is a product of principled design. Experimental results confirmed the effectiveness of this approach, with our model outperforming the baseline YOLOv8-Pose and other state-of-the-art lightweight models on key metrics for both object detection and pose estimation.

Despite these advances, the study has limitations. An analysis of failure cases revealed that in low-light conditions, the model can confuse the musk deer with the concrete background, an extreme case of the natural camouflage problem that generic data augmentation does not fully resolve. Furthermore, the dataset’s current scale and behavioral diversity may constrain generalization to broader wild or cross-farm scenarios. Targeted directions for future work thus include learning illumination-invariant features or integrating multimodal sensor data to achieve robust around-the-clock monitoring. We also aim to scale data collection across diverse ecological settings and incorporate domain adaptation and semi-supervised learning to reduce reliance on costly manual labeling. Through algorithmic optimization, we will further pursue deployment on edge devices to construct a real-time, automated, and intelligent monitoring system. This work ultimately offers a scientific and effective technical tool for the welfare-informed artificial breeding and data-driven conservation of the forest musk deer and other endangered species.

## Figures and Tables

**Table 1:** Forest musk deer keypoint annotation positions.

Number	Keypoint	Annotation Location
0	Nose tip	Center point of the tip of the nose
1	Left ear	Center point of the left ear on the head
2	Right ear	Center point of the right ear on the head
3	Neck	Center point of the connection between the head and trunk
4	Upper spine	Top vertex of the dorsal spine
5	Lower spine	Bottom endpoint of the dorsal spine
6	Left shoulder	Left shoulder position of the trunk
7	Left forelimb knee	Middle bending joint of the left forelimb
8	Left forelimb hoof	Ground contact point of the left forelimb end
9	Right shoulder	Right shoulder position of the trunk
10	Right forelimb knee	Middle bending joint of the right forelimb
11	Right forelimb hoof	Ground contact point of the right forelimb end
12	Left hip	Left hip position of the trunk
13	Left hindlimb knee	Middle bending joint of the left hindlimb
14	Left hindlimb hoof	Ground contact point of the left hindlimb end
15	Right hip	Right hip position of the trunk
16	Right hindlimb knee	Middle bending joint of the right hindlimb
17	Right hindlimb hoof	Ground contact point of the right hindlimb end

**Table 2:** Data volume of each behavior before the musk deer data augmentation.

Behaviors	Training Images	Validation Images	Total Images
Lying/sleeping	119	30	149
Standing/walking	402	101	503
Foraging	132	34	166
Defecation	104	26	130

**Table 3:** Data volume of each behavior in the musk deer dataset after data augmentation.

Behaviors	Training Images	Validation Images	Total Images
Lying/sleeping	595	150	745
Standing/walking	2010	505	2515
Foraging	660	170	830
Defecation	520	130	650

**Table 4:** Comparison experiment results on the forest musk deer behavior dataset.

Model	Box-mAP50	Box-mAP50-95	Pose-mAP50	Pose-mAP50-95	Parameters (M)	GFLOPs (G)	FPS
Keypoint R-CNN [36]	0.964	0.732	0.942	0.71	59.2	——	25.59
YOLOv8n-Pose	0.925	0.802	0.854	0.55	3.312	9.3	104.5
YOLOv11n-Pose	0.929	0.812	0.871	0.539	2.889	7.5	91.47
YOLOv12n-Pose	0.924	0.803	0.847	0.527	2.863	7.5	72.76
YOLO26n-Pose	0.925	0.79	0.884	0.594	2.972	7.7	95.46
Ours	0.929	0.814	0.879	0.565	3.66	9.6	90.61

Note: n denotes the nano version.

**Table 5:** Ablation experiment results on the forest musk deer behavior dataset.

YOLOv8n-Pose	DILATED-SPPF	MDSC-Mixer	Box-P	Box-mAP50	Pose-P	Pose-mAP50	Parameters (M)	GFLOPs (G)
√			0.893	0.925	0.875	0.854	3.312	9.3
√	√		0.915	0.919	0.875	0.868	3.509	9.4
√		√	0.926	0.932	0.881	0.864	3.463	9.4
√	√	√	0.906	0.929	0.897	0.879	3.66	9.6

Note: The symbol “√” represents that the corresponding experimental module is introduced and applied.

**Table 6:** Additional experiment results for the forest musk deer behavior dataset.

	Box-P	Box-mAP50	Box-mAP50-95	Pose-P	Pose-mAP50	Pose-mAP50-95
Experiment 1	0.916	0.925	0.802	0.882	0.869	0.554
Experiment 2	0.902	0.923	0.802	0.89	0.858	0.55
Experiment 3	0.905	0.929	0.814	0.897	0.879	0.564
Experiment 4	0.916	0.925	0.802	0.882	0.869	0.554

## Data Availability

The data presented in this study are available on request from the corresponding author. Due to commercial confidentiality and licensing agreements, the raw dataset is not publicly distributable.

## References

[1] Zheng C., Wu W., Chen C., Yang T., Zhu S., Shen J., Kehtarnavaz N., Shah M. (2023). Deep learning-based human pose estimation: A survey. ACM Comput. Surv..

[2] Rahman Z.U., Asaari M.S.M., Ibrahim H. (2025). Advancing animal farming with deep learning: A systematic review. Comput. Electron. Agric..

[3] Fang C., Zhang T., Zheng H., Huang J., Cuan K. (2021). Pose estimation and behavior classification of broiler chickens based on deep neural networks. Comput. Electron. Agric..

[4] Luo J., Zhu L., Li L., Hong P. (2023). Robot visual servoing grasping based on top-down keypoint detection network. IEEE Trans. Instrum. Meas..

[5] Ye S., Filippova A., Lauer J., Schneider S., Vidal M., Qiu T., Mathis A., Mathis M.W. (2024). SuperAnimal pretrained pose estimation models for behavioral analysis. Nat. Commun..

[6] Chen J., Liu L., Li P., Yao W., Shen M., Ding Q., Liu L. (2025). PKL-track: A keypoint-optimized approach for piglet tracking and activity measurement. Comput. Electron. Agric..

[7] Su L., Li M., Zhang Y., Zong Z., Gong C. (2024). Fusion of target and keypoint detection for automated measurement of Mongolian horse body measurements. Agriculture.

[8] Volkmann N., Zelenka C., Devaraju A.M., Brünger J., Stracke J., Spindler B., Kemper N., Koch R. (2022). Keypoint detection for injury identification during turkey husbandry using neural networks. Sensors.

[9] Jia Z., Zhao Y., Mu X., Liu D., Wang Z., Yao J., Yang X. (2025). Intelligent deep learning and keypoint tracking-based detection of lameness in dairy cows. Vet. Sci..

[10] Li J., Liu Y., Zheng W., Chen X., Ma Y., Guo L. (2024). Monitoring cattle ruminating behavior based on an improved keypoint detection model. Animals.

[11] Yang L., Jiang T., Gui X., Duan Q. (2024). Automated body measurement of beef cattle based on keypoint detection and local point cloud clustering. Meas. Sci. Technol..

[12] Peng C., Cao S., Li S., Bai T., Zhao Z., Sun W. (2024). Automated measurement of cattle dimensions using improved keypoint detection combined with unilateral depth imaging. Animals.

[13] Yang G., Qiao Y., Deng H., Shi J.Q., Song H. (2025). One-stage keypoint detection network for end-to-end cow body measurement. Eng. Appl. Artif. Intell..

[14] Zhao Y., Chen M., Feng G., Zhai W., Xiao P., Huang Y. (2025). Fine-grained fish individual recognition in underwater environments using global detail enhancement and keypoint region fusion. Fishes.

[15] Wu C., Zhang S., Wang W., Wu Z., Yang S., Chen W. (2025). Computation and analysis of phenotypic parameters of Scylla paramamosain based on YOLOv11-DYPF keypoint detection. Aquac. Eng..

[16] Chen K., Chen Z., Wang C., Zhou Z., Xiao M., Zhu H., Li D., Liu W. (2025). Improved YOLOv8-based method for the carapace keypoint detection and size measurement of Chinese mitten crabs. Animals.

[17] Sledevič T., Serackis A., Matuzevičius D., Plonis D., Andriukaitis D. (2024). Keypoint-based bee orientation estimation and ramp detection at the hive entrance for bee behavior identification system. Agriculture.

[18] Zhao X., Wu X., Miao J., Chen W., Chen P.C.Y., Li Z. (2022). Alike: Accurate and lightweight keypoint detection and descriptor extraction. IEEE Trans. Multimed..

[19] Zhao X., Wu X., Chen W., Chen P.C.Y., Xu Q., Li Z. (2023). Aliked: A lighter keypoint and descriptor extraction network via deformable transformation. IEEE Trans. Instrum. Meas..

[20] Zhang H., Xu L., Lai S., Shao W., Zheng N., Luo P., Qiao Y., Zhang K. (2024). Open-vocabulary animal keypoint detection with semantic-feature matching. Int. J. Comput. Vis..

[21] Chen S., Liao Y., Chen J., Lin F. (2024). Improved keypoint localization network for tea bud based on YOLO framework. Comput. Electr. Eng..

[22] Jiang T., Li Y., Feng H., Wu J., Sun W., Ruan Y. (2024). Research on a trellis grape stem recognition method based on YOLOv8n-GP. Agriculture.

[23] Deng L., Ma R., Chen B., Song G. (2025). A detection method for synchronous recognition of string tomatoes and picking points based on keypoint detection. Front. Plant Sci..

[24] Du X., Meng Z., Ma Z., Lu W., Cheng H. (2023). Tomato 3D pose detection algorithm based on keypoint detection and point cloud processing. Comput. Electron. Agric..

[25] Shen Y., Li B., Wang Y., Li Q., Zhang Z. (2025). An algorithm for detecting cow lameness based on ensemble learning of keypoint motion features. J. Dairy Sci..

[26] Yang Y., Deng Y., Li J., Liu M., Yao Y., Peng Z., Gu L., Peng Y. (2024). An effective yak behavior classification model with improved YOLO-pose network using yak skeleton key points images. Agriculture.

[27] Gong C., Zhang Y., Wei Y., Du X., Su L., Weng Z. (2022). Multicow pose estimation based on keypoint extraction. PLoS ONE.

[28] Yu M., Zhang L., Wei Y., Zhu L., Jiang X., Zhang J., Meng H., Lu Y. (2025). Automatic measurement method for sheep body dimensions based on posture compensation. Signal Image Video Process..

[29] Chen X., Guo X., Li Y., Liu C. (2025). A lightweight automatic cattle body measurement method based on keypoint detection. Symmetry.

[30] Li R., Wen Y., Zhang S., Xu X., Ma B., Song H. (2024). Automated measurement of beef cattle body size via key point detection and monocular depth estimation. Expert Syst. Appl..

[31] Peng Y., Peng Z., Zou H., Liu M., Hu R., Xiao J., Liao H., Yang Y., Huo L., Wang Z. (2024). A dynamic individual method for yak heifer live body weight estimation using the YOLOv8 network and body parameter detection algorithm. J. Dairy Sci..

[32] Yu F., Koltun V. (2015). Multi-scale context aggregation by dilated convolutions. arXiv.

[33] Wang X., Shi N., Wang G., Shao J., Zhao S. (2023). A multi-channel parallel keypoint fusion framework for human pose estimation. Electronics.

[34] Dutta H.P.J., Bhuyan M.K., Karsh R.K., Alfarhood S., Safran M. (2024). Multiscale attention-based hand keypoint detection. IEEE Trans. Instrum. Meas..

[35] Zhang J., Chen Z., Tao D. (2021). Towards high performance human keypoint detection. Int. J. Comput. Vis..

[36] He K., Gkioxari G., Dollár P., Girshick R. Mask R-CNN. Proceedings of the IEEE International Conference on Computer Vision (ICCV).

[37] Wang S. (2025). Domain adaptation using transformer models for automated detection of exterior cladding materials in street view images. Sci. Rep..

[38] Wang S. (2026). Domain-adaptive faster R-CNN for non-PPE identification on construction sites from body-worn and general images. Sci. Rep..

